# Supplementary material for: Novel TiO2 Nanoparticles/Polysulfone Composite Hollow Microspheres for Photocatalytic Degradation
Source: Polymers (Basel). 2021 Jan 21;13(3):336. doi: 10.3390/polym13030336 (PMC7865213; doi:10.3390/polym13030336)
Supplement: Supplementary file 1 [file polymers-13-00336-s001.pdf]

polymers

Supporting Information

Novel TiO_2 Nanoparticles/ Polysulfone Composite Hollow Microspheres for Photocatalytic Degradation

Shangying Zhang, Qi Wang, Fengna Dai, Yangyang Gu, Guangtao Qian, Chunhai Chen and Youhai Yu *

Center for Advanced Low-Dimension Materials, State Key Laboratory for Modification of Chemical Fibers and Polymer Materials, College of Materials Science and Engineering, Donghua University, Shanghai 201620, China; 1185099@mail.dhu.edu.cn (S.Z.); 1185078@mail.dhu.edu.cn (Q.W.); 2180397@mail.dhu.edu.cn (F.D.); 1209718@mail.dhu.edu.cn (Y.G.); qgt@dhu.edu.cn (G.Q.); cch@dhu.edu.cn (C.C.)

* Correspondence: yuyouhai@dhu.edu.cn

Table of contents

Figure S1 EDS of the inner surface of the TiNPs/PSF-0.40 composite microspheres.

Figure S2 (a) XPS full-scale spectra of TiO2, peak-fitting XPS of the (b) Ti 2p, (c) O 1s; (d) XPS full-scale spectra of PSF, peak-fitting XPS of the (e) C 1S, (e) S 2p.

Table S1. Adsorption capacity of TiO_2, PSF and TiNPs/PSF microsphere

Citation: Zhang, S.Y.; Wang, Q.; Dai, F.N.; Gu, Y.; Qian, G.T.; Chen, C.H. and Yu, Y.H. Novel TiO_2 Nanoparticles/Polysulfone Composite Hollow Microspheres for Photocatalytic Degradation. *Polymers* **2020**, *13*, x. https://doi.org/10.3390/xxxxx

Received: 29 December 2020
Accepted: 18 January 2021
Published: date

Publisher's Note: MDPI stays neutral with regard to jurisdictional claims in published maps and institutional affiliations.

Copyright: © 2021 by the authors. Licensee MDPI, Basel, Switzerland. This article is an open access article distributed under the terms and conditions of the Creative Commons Attribution (CC BY) license (http://creativecommons.org/licenses/by/4.0/).

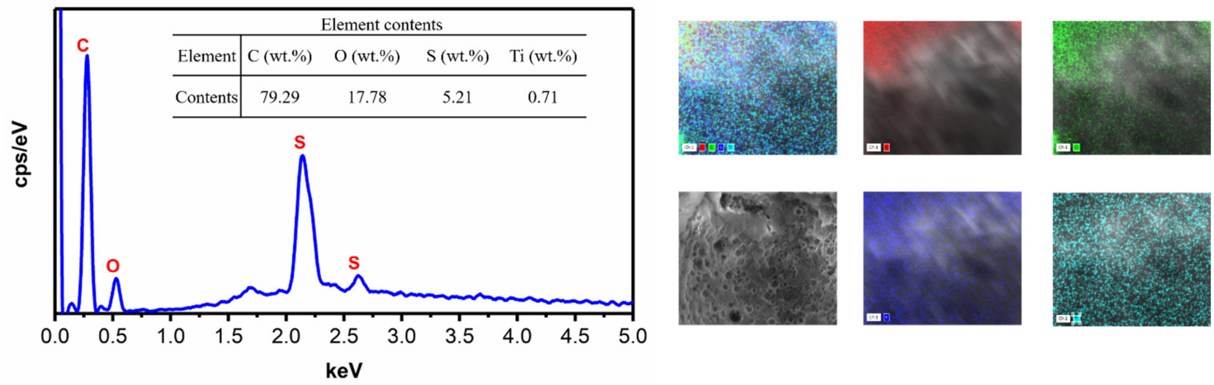

Figure S1. EDS of the inner surface of the TiNPs/PSF-0.40 composite microspheres.

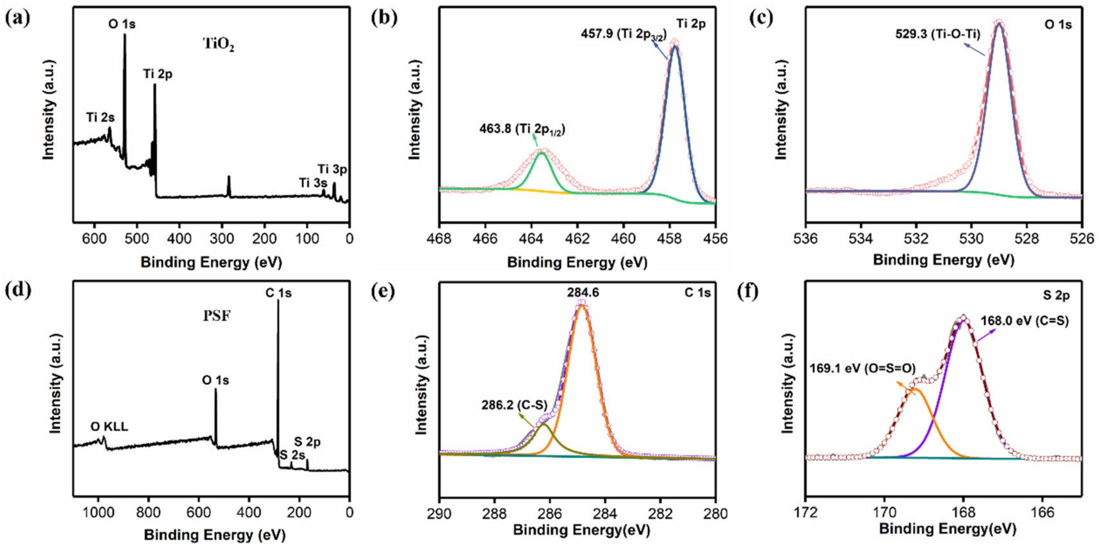

Figure S2. (**a**) XPS full-scale spectra of TiO_2, peak-fitting XPS of the (**b**) Ti 2p, (**c**) O 1s; (**d**) XPS full-scale spectra of PSF, peak-fitting XPS of the (**e**) C 1S, (**f**) S 2p.

Table S1. Adsorption capacity of TiO2, PSF and TiNPs/PSF microsphere.

Time / Sample	5 min (mg g^{-1})	10 min (mg g^{-1})	15 min (mg g^{-1})	20 min (mg g^{-1})	30 min (mg g^{-1})
pure PSF	0.28	0.59	0.62	0.65	0.65
TiO_2	0.12	0.33	0.56	0.78	0.78
TiNPs/PSF-0.08	0.33	0.65	0.70	0.71	0.71
TiNPs/PSF-0.24	0.35	0.69	0.74	0.76	0.77
TiNPs/PSF-0.40	0.36	0.68	0.73	0.77	0.77
TiNPs/PSF-0.56	0.38	0.7	0.74	0.79	0.79
TiNPs/PSF-0.72	0.35	0.68	0.71	0.75	0.76
TiNPs/PSF-0.88	0.33	0.68	0.72	0.73	0.74
